# Granulocyte transfusions in children and adults with hematological malignancies: benefits and controversies

**DOI:** 10.1186/s12967-015-0724-5

**Published:** 2015-11-16

**Authors:** Chiara Cugno, Sara Deola, Perla Filippini, David F. Stroncek, Sergio Rutella

**Affiliations:** Division of Translational Medicine, Clinical Research Center, Sidra Medical and Research Center, Out-Patient Clinic, Al Luqta Street, Education City North Campus, P.O. Box 26999, Doha, Qatar; Department of Pediatric Hematology and Oncology, IRCCS Fondazione Policlinico San Matteo, Pavia, Italy; Hematology and Bone Marrow Transplant Unit, Ospedale Centrale Bolzano, Bolzano, Italy; Deep Immunophenotyping Core, Division of Translational Medicine, Sidra Medical and Research Center, Doha, Qatar; Cell Processing Section, Department of Transfusion Medicine, National Institutes of Health (NIH) Clinical Center, Bethesda, MD USA

**Keywords:** Granulocyte transfusion, G-CSF, Dexamethasone, Hematopoietic stem cell transplantation, Febrile neutropenia, Infection

## Abstract

Bacterial and fungal infections continue to pose a major clinical challenge in patients with prolonged severe neutropenia after chemotherapy or hematopoietic stem cell transplantation (HSCT). With the advent of granulocyte colony-stimulating factor (G-CSF) to mobilize neutrophils in healthy donors, granulocyte transfusions have been broadly used to prevent and/or treat life-threatening infections in patients with severe febrile neutropenia and/or neutrophil dysfunction. Although the results of randomized controlled trials are inconclusive, there are suggestions from pilot and retrospective studies that granulocyte transfusions may benefit selected categories of patients. We will critically appraise the evidence related to the use of therapeutic granulocyte transfusions in children and adults, highlighting current controversies in the field and discussing complementary approaches to modulate phagocyte function in the host.

## Background

Bacterial and fungal infections remain a significant cause of morbidity and mortality in severely neutropenic patients with hematological malignancies receiving dose-intensive chemotherapy and hematopoietic stem cell transplantation (HSCT). Once in vogue in the 1980s, granulocyte transfusions were later relegated to a marginal role, due to the inconveniences of harvesting cells, infusion-associated toxicities and limited clinical efficacy [1]. Several articles offer a historical perspective on granulocyte transfusions (GTX) and the reader is referred to those studies for a thorough overview [1–4]. The improved odds of survival shown by clinical trials conducted in the 1970s and 1980s may have been related to low survival rates of controls, implying that the benefit of adding granulocyte transfusions to contemporary treatment protocols for life-threatening infections may be questionable [5]. In the early 1990s, the clinical use of granulocyte colony-stimulating factor (G-CSF) and the advent of leukapheresis reignited the interest in the clinical application of granulocyte transfusions to enhance host defenses [3, 6–11]. However, inconsistent clinical responses have been documented over the years, likely due to differences in patient selection, underlying disorder, indications for granulocyte transfusion, e.g., prevention or treatment of infections, type of granulocyte concentrates, e.g., related vs. HLA-matched unrelated donors, use of G-CSF alone or G-CSF and steroids for donor priming, pre-existing alloimmunization or de novo development of antibodies against granulocytes, availability of donors, and treating physicians’ preference [12].

The results of new randomized trials of granulocyte transfusions for life-threatening infections have now been published. Also, new-generation leukapheresis devices have been optimized to further improve the yield and purity of granulocyte collections. It thus is an appropriate time to review existing data on granulocyte transfusions in neutropenic hosts, and to critically appraise the immune-biological effects and safety issues related to G-CSF administration to healthy donors. The following PubMed query was used to retrieve relevant clinical trials: (infection OR sepsis) AND (neutropenia OR granulocytopenia) AND (“granulocyte transfusion” OR “granulocyte transfusions”). We also searched both the U.S. National Institutes of Health registry of clinical studies (http://www.ClinicalTrials.gov) and the International Clinical Trials Registry Platform (ICTRP; http://apps.who.int/trialsearch/).

## Neutrophil mobilization

### G-CSF alone or in combination with steroids

The effects of single-dose G-CSF (5–10 µg/kg) on peripheral blood counts have been explored in 261 normal subjects donating peripheral blood progenitor cells (PBPC) for research. Following G-CSF administration, the neutrophil count increased 6.2- to 7.4-fold over baseline values, depending on G-CSF dose. Sixty-nine percent of the donors experienced one or more dose-related side effects 24 h after G-CSF administration, most commonly muscle and bone pain, headache, fatigue and nausea [13].

The combination of G-CSF and dexamethasone has been extensively characterized as a granulocyte mobilization regimen. Neutrophilia after single-dose G-CSF and dexamethasone is attributed to shifts of neutrophils from the bone marrow storage pool into the peripheral blood [14]. The analysis of a computerized database containing records of 1198 granulocyte collections from 137 unrelated volunteer apheresis donors showed that donors with higher neutrophil and platelet counts before stimulation have higher neutrophil counts after G-CSF and dexamethasone. A uniform G-CSF dose of 480 μg was equally effective as weight-based dosing at 5 µg/kg [15]. In another study, 20 donors received oral dexamethasone (8 mg) plus a placebo injection, subcutaneous G-CSF (5 μg/kg) plus placebo capsules, or G-CSF plus dexamethasone. The administration of G-CSF plus dexamethasone produced the greatest yields and was not associated with increased toxicity as compared with G-CSF alone [16].

In a prospective study, 52 healthy unrelated volunteers were treated with a single subcutaneous injection of glycosylated G-CSF, lenograstim, at a median dose of 3.1 μg/kg plus dexamethasone (8 mg orally) or with a median dose of 11.8 μg/kg of lenograstim without dexamethasone (n = 23). Mobilization kinetics and leukapheresis yields were similar in the low-dose compared with the high-dose G-CSF group. Donor adverse reactions were of greater clinical significance in donors given high-dose G-CSF alone. The combination of glycosylated G-CSF and dexamethasone allowed a significant reduction of G-CSF dose and enhanced the tolerability of the mobilization regimen to the donors [17].

Another study evaluated the efficacy of four different granulocyte mobilization regimens. Donors received G-CSF, either intravenously or subcutaneously, with or without dexamethasone (8 mg) 18 h before apheresis. Whereas G-CSF administration route had no impact on neutrophil counts at hours 2 through 8, subcutaneous G-CSF and dexamethasone sustained 24-h neutrophil counts more effectively than intravenous G-CSF and dexamethasone [18].

A possible strategy to minimize the number of donors for each patient, thus limiting the risk of alloimmunization, would be to use sequential granulocyte collections from a single donor given G-CSF daily. This approach has been prospectively evaluated in 76 healthy donors, who were allowed a maximum of five consecutive donations [19]. This mobilization schedule translated into a continuing increase of white blood cells and neutrophils, leading to better collection yields. The side effects related to repeat administrations of G-CSF were tolerable, not exceeding WHO grade II status. Bone pain, headache, arthralgia, and myalgia were commonly observed (24 % of the donors), but were transient and responsive to paracetamol. Donors who underwent multiple leukapheresis procedures gained a median of 1.0 kg of body weight, and developed minimal peripheral edema. In one donor, a mild skin reaction, likely due to HES, was documented on the third day of granulocyte donation [19].

### Impact of the mobilization regimen on neutrophil phenotype and function

G-CSF has long been recognized as a potent immediate activator of neutrophils in vivo [20]. An initial study in four healthy volunteers given subcutaneous G-CSF (300 µg) showed that, shortly post-injection, CD16 was upregulated from an intracellular pool. Specific granules were released, as suggested by increased plasma levels of lactoferrin and by upregulation of CD66b and CD11b expression on circulating neutrophils. Moreover, increased plasma levels of elastase, bound to its physiologic inhibitor α1-antitrypsin, indicated mobilization of azurophil granules [20].

The expression of neutrophil antigens has been thoroughly evaluated in seven healthy donors receiving 5 μg/kg of G-CSF for 10 days [21]. The expression of l-selectin (CD62L), Fcγ receptor (FcγR) III (FcγRIII, CD16), and the leukocyte function antigen (CD11a) decreased during the course of G-CSF administration. By contrast, FcγRI (CD64) and lipopolysaccharide-binding protein receptor (CD14) levels increased. The expression of amino-peptidase N (CD13), C3bi receptor (CD11b), and the neutrophil β2-integrin (CD18) did not change during the administration of G-CSF, but levels of both CD13 and CD18 increased 3 days after the last dose. Expression levels of neutrophil-specific antigen NB1 (CD177) initially increased, returned to pre-G-CSF levels after 4 days, and increased again after 10 days of G-CSF administration. Overall, this study indicated that most changes in neutrophil phenotype, potentially affecting the function of mobilized granulocytes, occur after one dose of G-CSF. Importantly, the down-regulation of CD62L after G-CSF treatment may result from shedding and likely limits neutrophil-endothelial cell interactions, thus preventing the pulmonary sequestration of granulocytes [21, 22].

Oligonucleotide microarrays were also used to identify genes that are differentially expressed before and after mobilization with single-dose G-CSF and dexamethasone [23]. More than 1000 genes displayed a differential expression pattern and, among these, many encoded proteins involved in inflammation and the immune response, such as C-type lectins and leukocyte immunoglobulin-like receptors. These changes could only partly be mimicked by in vitro culture of normal granulocytes with 100 ng/mL G-CSF and 1 μM dexamethasone, since more than 75 % of changes in gene expression were unique to in vivo mobilization with the two drugs. Interestingly, transcriptional activity of the *CAST* gene, which encodes calpastatin, was induced after G-CSF/dexamethasone treatment both in vivo and in vitro. This observation may account for the prolonged survival ability imparted on granulocytes by the combined treatment with G-CSF and dexamethasone. Irrespective of phenotypic changes, granulocytes collected after G-CSF and dexamethasone benefited 11 out of the 16 children (70 %) who were treated with granulocyte transfusions for febrile neutropenia [24].

**Fig. 1:**
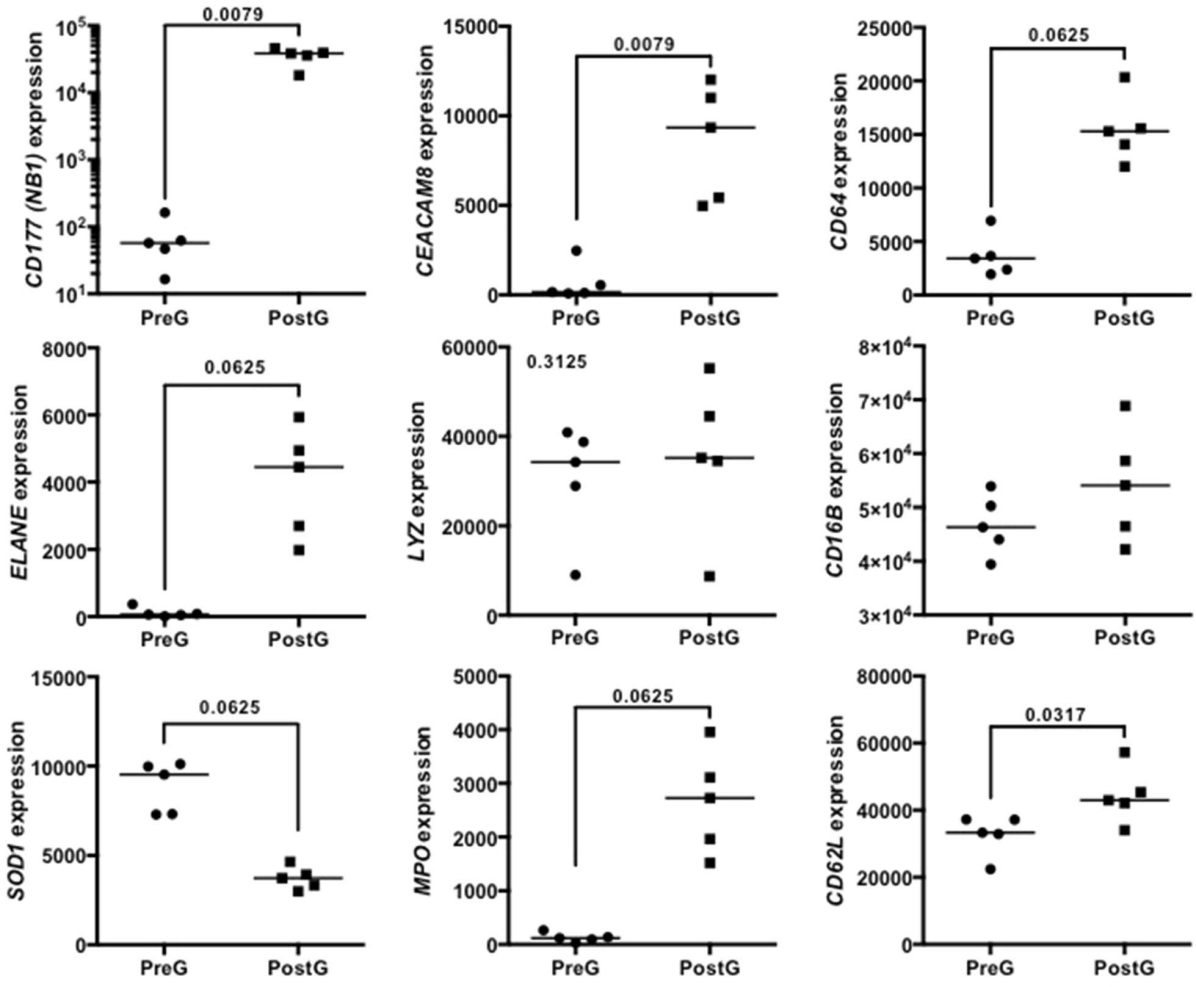
Abundance of selected transcripts in leukocytes from donors receiving G-CSF. This dataset (http://www.ncbi.nlm.nih.gov/sites/GDSbrowser?acc=GDS2959) was selected among studies currently available in NCBI’s Gene Expression Omnibus (GEO; http://www.ncbi.nlm.nih.gov/sites/GDSbrowser). In curated datasets, the ‘Data Analysis Tools’ button allows the user to gain access to gene expression levels by directly providing the gene name or symbol. Within non-curated datasets, the GEO2R tool allows the user to select a gene platform and to compare two or more groups of samples in order to identify genes that are differentially expressed across experimental conditions. This study analyzed the genome-wide patterns of gene expression with DNA microarrays (Affymetrix Human Genome U133 Plus 2.0 Array) in five healthy donors given G-CSF [25]. The expression levels of antigens relevant for granulocyte function are shown. Data were compared with the Mann–Whitney *U* test. A *p* value <0.05 was considered to denote statistical significance

**Fig. 2:**
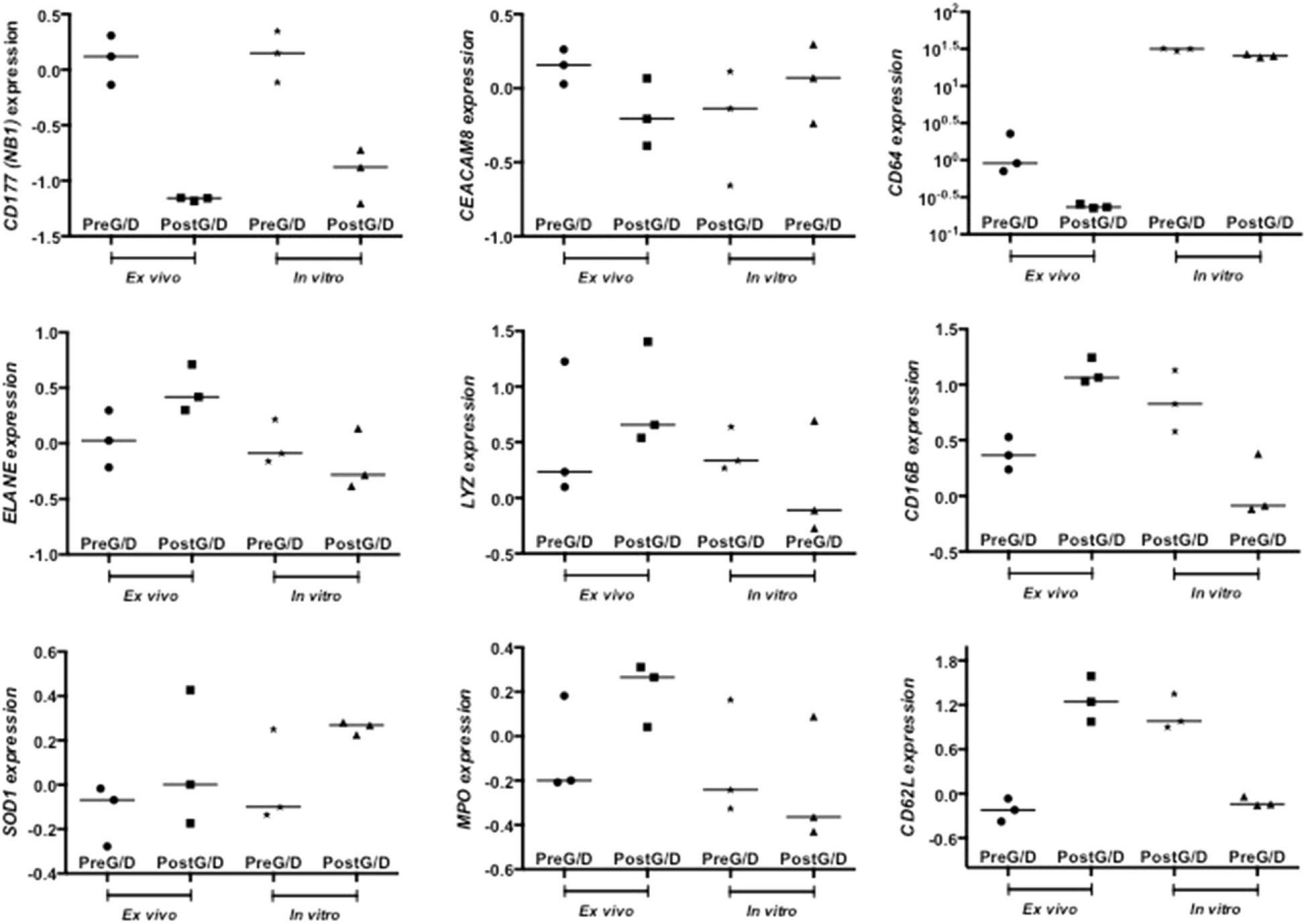
Abundance of selected transcripts in leukocytes from donors receiving G-CSF and dexamethasone. This dataset was selected among studies currently available in NCBI’s Gene Expression Omnibus (GEO; http//www.ncbi.nlm.nih.gov/sites/GDSbrowser?acc=GSE12841). Granulocytes were isolated from three individuals before and 18 h after treatment with G-CSF and dexamethasone. Some of the control cells were cultured overnight in HBSS medium with or without the addition of G-CSF and dexamethasone. Total RNA from each experimental condition was compared to pooled RNA of control granulocytes. The genome-wide pattern of gene expression was analyzed with DNA microarrays (Agilent-012391 Whole Human Genome Oligo Microarray G4112A) [23]. Data were compared with the Mann–Whitney *U* test. A *p* value <0.05 was considered to denote statistical significance

Finally, we pursued a data mining approach to navigate publicly available datasets generated in the context of G-CSF administration to healthy donors, with the aim at identifying differences in transcriptomic profiles of leukocytes from subjects given G-CSF alone or in combination with steroids. We retrieved three datasets containing expression profiling data of leukocytes and monocytes from healthy donors receiving G-CSF alone (n = 2) or G-CSF and dexamethasone (n = 1; http://www.ncbi.nlm.nih.gov/gds). We identified a priori a panel of genes regulating neutrophil effector functions (Table 1) and we asked whether their expression was modulated across different datasets available from the literature. As shown in Fig. 1, the abundance of mRNA transcripts for *CD177*, *ELANE*, *MPO*, *LYZ*, *CEACAM8* (*CD67*), *CD64*, *CD16b*, and *CD62L* increased after donor treatment with G-CSF [25]. By contrast, *SOD1* mRNA expression declined after neutrophil mobilization. Compared with G-CSF alone, G-CSF and dexamethasone induced a decrease of *CD177* and *CD64* mRNA expression (Fig. 2). Interestingly, in vitro exposure of normal neutrophils to G-CSF and dexamethasone resulted in a different transcription expression profile of our genes of interest, compared with in vivo treatment. Specifically, in vitro-treated neutrophils failed to up-regulate *CD16b*, *CD62L*, *ELANE*, *MPO* and *LYZ* mRNA (Fig. 2), suggesting that neutrophil phenotype is uniquely influenced by in vivo exposure to G-CSF and dexamethasone. Collectively, G-CSF and dexamethasone provide advantages over G-CSF alone in terms of neutrophil mobilization efficiency and collection yields. However, further studies are needed to obtain insights into the functional changes induced in neutrophils by G-CSF in combination with steroids.

**Table 1:** Selected list of genes regulating neutrophil function

Gene	Role(s) in neutrophil homeostasis
*CD177*	NB1 is a glycosylphosphatidylinositol (GPI)-anchored glycoprotein expressed exclusively by neutrophils, metamyelocytes and myelocytes
*SOD1*	The SOD1 gene encodes superoxide dismutase-1, a major cytoplasmic antioxidant enzyme that metabolizes superoxide radicals to molecular oxygen and hydrogen peroxide. CD177 is upregulated in various inflammatory settings, including bacterial infections. Heterophilic PECAM1/CD177 interactions affect the phosphorylation state of PECAM1, as well as endothelial junction integrity and neutrophil transmigration
*CD64*	CD64 is the gene encoding human FcγRI (FCGR1), a glycoprotein that is constitutively expressed on human monocytes and macrophages and that plays a pivotal role in the immune response
*FCGR3B*	The Fc receptor with low affinity for IgG (CD16b) encodes a glycosylphosphatidylinositol (GPI)-anchored protein that is expressed constitutively by neutrophils
*ELANE*	Neutrophil elastase is a serine protease of neutrophil and monocyte granules, with key physiologic roles in innate host defense
*MPO*	Myeloperoxidase is a lysosomal protein located in the azurophilic granules of polymorphonuclear leukocytes and monocytes. MPO is responsible for microbicidal activity against a wide range of organisms
*LYZ*	Lysozyme catalyzes the hydrolysis of certain mucopolysaccharides of bacterial cell walls
*CEACAM8 (CD67)*	Neutrophil surface protein that is attached to the membrane via a glycosylphosphatidylinositol anchor. In neutrophils, the *CEACAM8* gene is primarily detected in the secondary cytoplasmic granules, but it can also be found in lower amounts on the plasma membrane. The amount of surface CD67 is upregulated upon granulocyte activation
*CD62L*	Homing receptor for lymphocytes to enter secondary lymphoid tissues via high endothelial venules

Based on current knowledge, a panel of genes was selected that have been shown to contribute to neutrophil function in health and/or disease states. A brief description of the main function of each gene product in neutrophil homeostasis is provided

## Granulocyte collection and storage

Granulocytes for transfusion are usually produced by either apheresis or as a component derived from whole blood donations [26]. Apheresis allows for the collection of large quantities of granulocyte from a single donor over a few hours. In order to obtain similar quantities of granulocytes from whole blood donations, granulocytes obtained from many units of blood must be pooled. For apheresis collections, the use of high molecular weight hydroxyethyl starch (HES) has been shown to result in better granulocyte collection yields, with decreased contamination from red blood cells and platelets in the granulocyte concentrate [27]. The use of blood cell separators to collect granulocytes concentrates by apheresis from subjects given G-CSF or G-CSF plus dexamethasone has become the standard. However, since there are practical difficulties and regulatory requirements for hospitals providing apheresis granulocyte components on demand, some groups have recently re-evaluated the use of granulocyte components derived from whole blood, which can be used as a bridging therapy while identifying suitable apheresis donors, administering mobilizing agents and collecting granulocytes by apheresis [26]. A standard adult granulocyte component can be derived from 20 whole blood donations, providing a daily dose of approximately 2 × 10^10^ granulocytes. The adverse events in recipients of granulocytes prepared with this approach appear to be comparable to those of recipients of other granulocyte components [26].

### Cell separation devices

A recent prospective, multicenter, randomized trial compared the performance and safety of a novel granulocyte collection protocol with the Spectra Optia^®^ device (Terumo-BCT) to those of the COBE Spectra apheresis system (Terumo-BCT) in 32 healthy donors, who received G-CSF and dexamethasone. The collection efficiency of granulocyte procedures using the Spectra Optia^®^ system was approximately 23 % higher than that of the COBE Spectra system. In particular, Spectra Optia^®^ collections generated a higher total neutrophil yield per liter of blood processed compared with the COBE Spectra. No differences in granulocyte viability, chemotaxis and bacterial killing were evident between the two devices. Not unexpectedly, fewer operator adjustments were required when using the Spectra Optia^®^. Importantly, there was no difference in the number or intensity of adverse donor events between instruments [28].

The Optia device has also been used to collect granulocytes from healthy blood donors and family donors mobilized with prednisolone or G-CSF. [29]. One study showed that the Optia^®^ device is not inferior to the COBE Spectra forerunner and produces high-quality granulocyte concentrates with greater absolute neutrophil yields. In another study, target doses of 1 × 10^10^ neutrophils were achieved in all but one collection with the Spectra Optia^®^ device [30]. Again, Spectra Optia^®^ collections were 20 % more efficient compared with those performed with the predecessor device.

Since the dose of neutrophils transfused is important in determining clinical response, the ability to collect higher numbers of granulocytes will favorably impact the efficacy of therapeutic granulocyte transfusions.

### Neutrophil storage

Although G-CSF and dexamethasone delay granulocyte apoptosis, the high cell counts achieved in granulocyte concentrates may reduce nutrients and lower pH, resulting into neutrophil death. The production of pyrogenic cytokines may also be increased. According to current standards, granulocyte storage should be limited to 24 h. After 2 days of storage in RPMI-1640 medium at 4 °C, only 2–7 % of the granulocytes remain viable [31]. Infusible solutions to be used in place of autologous plasma have been designed and tested with the aim of improving granulocyte storage. For instance, lactated Ringer’s solution or Plasma-Lyte A supplemented with buffers and albumin hold promise as effective and licensable solutions for granulocyte storage [32].

The storage characteristics of granulocyte concentrates were compared after mobilization of donors with G-CSF and dexamethasone, either alone or in combination [33]. Ten granulocyte donors were given oral dexamethasone (8 mg), subcutaneous G-CSF (5 μg/kg), or both and granulocyte concentrates were collected by leukapheresis on the next day. Significantly more granulocytes were collected from donors given G-CSF or G-CSF + dexamethasone compared with dexamethasone alone. Granulocytes mobilized with G-CSF + dexamethasone were acidic immediately after collection, with pH values declining below 6.0 after 24 h. Some increase in IL-1β and IL-8 was detected after 24 and 48 h as compared to the levels at 2 h storage. By contrast, levels of IL-6 and TNF were unchanged. Serial dilutions in autologous plasma were also performed on 13 granulocyte concentrates prior to storage. Importantly, the pH remained above 7.0 only when dexamethasone-mobilized granulocytes were diluted 1-in-8, and when G-CSF + dexamethasone-mobilized granulocytes were diluted 1-in-16, an operationally impractical condition given the high volume of autologous plasma needed. This study strongly suggests that clinical-grade granulocyte preservative solutions are needed to maintain pH during storage.

Neutrophil chemotaxis and NADPH oxidase activity were also examined after apheresis collection and neutrophil storage to 48 h [34]. After in vivo mobilization with G-CSF, neutrophils were collected and stored in apheresis bags, with or without additional G-CSF. Neutrophil baseline chemotaxis and NADPH activity were preserved to 24 h of storage and were not affected by exogenously added G-CSF, indicating that biochemical integrity for oxidase activity is maintained during short-term storage.

Overall, commercial leukocyte storage solutions can prolong granulocyte survival up to 7 days. However, testing of granulocytes with monoclonal antibodies or alloantibodies is problematic after 4 days due to nonspecific staining.

## Clinical trials of granulocyte transfusions

### Children

Infections in neutropenic patients are associated with a severe prognosis, despite treatment with broad-spectrum antibiotics. In children with malignancy and septic shock, mortality can be greater than 40 % [35] or even higher, up to 85 %, in specific patient subpopulations, such as bone marrow transplant recipients with invasive aspergillosis [8]. Use of G-CSF and recent improvements in apheresis techniques allow the collection of large quantities of granulocytes, which result in increases in granulocyte counts in the transfusion recipients, particularly in children [36]. The main criteria for the clinical use of GTX in children have included the presence of severe neutropenia with absolute neutrophil counts <0.5 × 10^9^/L [37–41], 0.2 × 10^9^/L [42] or 0.1 × 10^9^/L [43], associated with documented severe infection, not responsive to broad-spectrum antibiotics and/or antifungal treatments. Published and ongoing clinical trials in children are summarized in Table 2.

**Table 2:** Clinical trials in children

# of pts	Clinical trial	Indications for GTX	Remarks/outcome	Reference(s)
27	Prospective, phase II	Severe neutropenia and infections	Donor mobilization: 7.5 μg/kg G-CSF; resolution of infection in 92.6 % of patients; 81.5 % OS on day +30; early administration after a median infection period of 6 days	[41]
49	Prospective	Neutropenia and invasive bacterial or fungal infection	Donor mobilization with 5 μg/kg G-CSF + 50 mg PDN. Mixed cohort, including 10 adults; 72 % OS on day +28 and 52 % OS on day +100	[42]
13	Prospective phase I/II	Neutropenia and severe infection	Donor mobilization with 5–10 μg/kg G-CSF; Collection through the bag method. 69 % OS on day +30	[90]
3	Prospective	CGD and invasive aspergillosis	Donor’s mobilization with 450 μg G-CSF + 8 mg DXM; one patient died for ARDS, one was lost at follow-up and died 1 year after discharge, one is alive	[49]
35	Retrospective	Febrile neutropenia or defective granulocyte function	Donor mobilization with 480 μg G-CSF + 8 mg DXM; OS 77.1 and 65.7 %, respectively, on day +30 and +60; 82.4 % infection-related OS	[40]
32	Retrospective	Sepsis and neutropenia	Donor mobilization with single-dose lenograstim + DXM 8 mg; 59 % OS (8/32 pts died for the underlying infection and 8/32 pts for non-infectious causes)	[39]
16	Retrospective	Severe neutropenia and documented bacterial and/or fungal infections in HSCT recipients	Donor mobilization with 8 mg DXM after 2007; unstimulated donors before 2007; 50 % OS on day +30	[37]
10	Retrospective	High risk febrile neutropenia with/without microbiologically documented severe infection	Donor mobilization with 5 μg/kg G-CSF + 8 mg DXM; Clinical response rate 62.9 %, 40 % infection-related mortality, 40 OS %	[91]
13	Retrospective	Febrile neutropenia	Resolution of the documented infection in 9/12 (75 %) pts; good early survival (12/14 courses of GTX, 86 %); poor long-term survival (5/13 pts, 39 %)	[47]
13	Retrospective	Severe infections and neutropenia	Donor mobilization with G-CSF 300 μg from day -3; complete or partial recovery in 6 and 3 of the 15 courses of GTX (40 and 20 % respectively)	[43]
13	Retrospective	Granulocyte dysfunction or severe neutropenia and acute life-threatening infections	Donor mobilization with 600 μg G-CSF + 8 mg DXM; complete or partial clinical response in 12/13 pts (92 %); 15 % infection-related mortality and 42 % OS	[38]
3	Retrospective	Secondary prophylaxis of invasive fungal infections during neutropenic episodes	Donor mobilization with G-CSF; concomitant combination antifungal therapy; no infection-related mortality	[39]
3	Prospective	Prophylaxis in HSC recipients with chronic infections	Donor mobilization with 480 μg G-CSF + 7.5 mg DXM; after transplant, no flares of the infections (active *S. aureus* liver abscesses, chronic pulmonary aspergillosis, soft tissue mucormycosis)	[50]
20	Prospective	Proven fungal or bacterial infection, unresponsive to anti-microbial therapy (n = 16). Poor control of fungal infection prior to allogeneic HSCT (n = 4)	In the curative group, infection was controlled in 11 out of 16 children. All patients treated pre-emptively survived the HSCT procedure	[24]
10	Prospective	CGD with severe infections	Resolution of infection in 9 out of 10 patients, despite the fact that 8 patients were alloimmunized and had poor recovery of transfused granulocytes	[65]

Completed and ongoing clinical trials of therapeutic granulocyte transfusions in children are summarized

*HSCT* hematopoietic stem cell transplantation, *DXM* dexamethasone, *OS* overall survival, *GTX* granulocyte transfusions, *CGD* chronic granulomatous disease

To date, there have been no randomized controlled GTX trials in children, and most available data are derived from observational studies [44]. Sachs et al. assessed the feasibility, safety and efficacy of early-onset G-CSF-mobilized GTX in an open, single-center, and prospective phase II clinical trial in immune-compromised children with neutropenia and severe infections, who failed to respond to broad-spectrum antibiotics [41]. The study utilized granulocytes collected from community donors and which were crossmatch compatible with the recipients’ serum. Twenty-seven children at high risk of infection-related mortality were treated between 2000 and 2004. Some patients also received either G-CSF or GM-CSF. GTXs were well tolerated, without any pulmonary transfusion reactions due to alloimmunization. Twenty-five of 27 patients cleared their initial infection. All six patients with invasive aspergillosis showed clinical and radiological improvement [41]. A noteworthy finding in this study was the remarkable response rate, probably due to the early initiation of GTX, i.e., after a median infection period of 6 days (range 3–18 days), compared with 8 days (range 1–28 days) [45], 12 days (range 2–36 days) [46], and 12 days (range 5–28 days) in other studies [43].

A retrospective analysis including 13 children with neutropenia and proven or suspected infection also supports the efficacy of granulocyte transfusions. Although short-term survival was promising, eight of the 13 patients ultimately died of their infection [47]. In another study of 35 children with high-risk febrile neutropenia or with granulocyte function defects, GTX were given for 3 consecutive days. The mean granulocyte content per concentrate was 27.4 × 10^9^. Infection-related survival and overall survival rates were 82 and 77 %, respectively, at day 30 [40]. Another retrospective study in children with febrile neutropenia or defective granulocyte function [40], who were given GTX for 3 consecutive days, showed overall survival rates of 77 and 63 %, respectively, at day +30 and +90 after GTX.

A 59 % overall survival rate was obtained in a cohort of 32 children, with particularly favorable results in bacterial infections (8/11 patients with documented bacterial infection survived) and fungal infection (4/6 patients with documented fungal invasive infection survived) [39]. In another case series, 13 children with sepsis who received 14 courses of GTX were reported to have a good short-term survival (12/14 courses, 86 %), whereas long-term outcome remained dismal (5/13 patients, 39 %) [47].

Seidel et al. [42] showed that neither body weight nor granulocyte dose impacted on infection outcome and survival in pediatric patients. Nonetheless, this study suggested that a tight schedule with daily transfusions of at least 1.4 × 10^8^ granulocytes/kg likely contributed better clinical outcomes. This minimum recommended dose was derived from a Cochrane meta-analysis [48]. They also reported the effect of daily GTX over at least 5 days containing a minimum of 3 × 10^8^/kg neutrophils per concentrate was able to generate a stable ANC increment, to shorten the duration of neutropenia, and to support the control of infections in neutropenic patients with high-risk infections.

Granulocyte transfusion therapy has been used in three patients with chronic granulomatous disease (CGD) and disseminated invasive aspergillosis. Healthy donors were mobilized with 450 µg G-CSF and dexamethasone approximately 12 h before collection. Patients received between 0.4 and 3.0 × 10^9^/kg granulocytes. Two out of three patients survived the infectious episode [49].

Some studies also suggest a role for granulocyte transfusions in preventing infections or progression of infections in children who are expected to experience prolonged neutropenia after HSCT or chemotherapy [39, 50]. Granulocyte transfusions from family volunteers were used prior to allogeneic HSCT in three children with poorly controlled bacterial or fungal infections. No transfusion-related reactions and no flares of the infection were observed. All HSCT procedures were successful [50].

Concern for potentially serious pulmonary complications is one of the major limiting factors for the routine use of GTX. Some studies of GTX recipients have documented acute pulmonary transfusion reactions with shortness of breath, dyspnea, hypoxemia, and lung edema [38, 43, 45, 51]. In a Cochrane meta-analysis (see also below), adverse events occurred in 15 % of the transfusions that had been collected by apheresis, but no reactions occurred in pre-medicated patients receiving granulocytes collected by apheresis. [48]. Moreover, the procedure of HLA-matching of the granulocyte donor and GTX recipient in a tight schedule of therapeutic GTX, as in Seidel’s prospective study [42], might carry the disadvantage of delayed treatment or lower granulocyte dosage.

Although randomized controlled trials are not available in children yet, the current evidence supports the early use of GTX, especially for patients with bacterial infections. However, patients should be closely monitored for adverse pulmonary transfusion reactions.

### Adults

Published and ongoing clinical trials in adults are summarized in Table 3. A meta-analysis published in 1997 reviewed eight randomized controlled trials conducted between 1970 and 1995 and were designed to assess the efficacy of prophylactic granulocyte transfusions [5]. The results suggested that daily prophylactic transfusions of compatible granulocytes could reduce the risk of bacterial or fungal infection, death or death from infection in patients with severe neutropenia. The study found that both granulocyte dose and granulocyte compatibility were determinants of the efficacy of granulocyte transfusions.

**Table 3:** Clinical trials in adults

# of pts	Study type	Indications for GTX	Remarks/outcome	Reference(s)
22	Retrospective	Grade IV febrile neutropenia	G-CSF only for neutrophil mobilization; when >10^10^ PMNs were infused, clinical benefit compared with historical controls	[89]
11	Case series	Invasive *Fusarium* infection	Ninety-one percent response rate	[57]
74	Retrospective	Treatment of infections	In 34 patients (46 %), GTXs were discontinued due to clinical response and neutrophil count recovery	[12]
56	Retrospective	Severe infection in SAA	GTX + G-CSF; Survival at 30, 90 and 180 days was 89, 70 and 66 %, respectively. Survival rate correlated with hematopoietic recovery	[58]
24	Retrospective	Invasive opportunistic infections	GTX + IFN-γ1b + G-CSF or GM-CSF. 60 % ORR 4 weeks after treatment	[87]
25	Prospective	Progressive uncontrolled infections	Donors given G-CSF and dexamethasone, either alone or in combination. Favorable responses in 40 % of patients (especially in those with fungal or Gram-negative infections). One death from severe pulmonary reaction	[51]
20	Pilot	Neutropenia refractory to G-CSF	Favorable response in 8 out of 15 assessable patients (53 %)	[53]
19	Phase I/II	Infections after HSCT	GTXs from community donors (94 %). G-CSF + dexamethasone. Resolution of infection in 8/19 patients (42 %). Overall, four of the 19 patients were alive on day 30 after HSCT. None of the patients with invasive aspergillosis (n = 5) cleared the infection	[52]
30	Retrospective	Neutropenia and severe infections	G-CSF + dexamethasone. In 11 patients, resolution of infection could be related to granulocyte transfusions. Three of these patients became long-term survivors	[54]
52	Prospective	Control or prevention of severe infections	Control of infections was achieved in 82 % of life-threatening episodes. No reactivation of infections occurred under prophylactic granulocyte transfusions	[55]
100	Randomized (GRANITE study)	Febrile neutropenia	Ongoing national, multi-center trial; Patients aged 1–75 years (www. drks.de/DRKS00000218); Date of first enrollment: October 2014; Arm 1 (intervention-group): transfusion of standardized leukapheresis products of granulocytes on every other day + standard therapy; arm 2 (control group): standard-therapy without granulocyte transfusions	NA
30	Prospective (GIN1 study)	Febrile neutropenia	Granulocytes derived from whole blood; risk of adverse events comparable to other granulocyte components; recovery of neutrophils and survival in all patients except for two adult patients who died	[26]
114	Randomized (RING study)	Febrile neutropenia	Composite endpoint was survival + microbial response 42 days after randomization; 42 and 43 % success rates for the granulocyte and control groups, respectively	NCT00627393; [56]

Completed and ongoing clinical trials of therapeutic granulocyte transfusions in adults are summarized

*HSCT* hematopoietic stem cell transplantation, *DXM* dexamethasone, *OS* overall survival, *ORR* overall response rate

A community blood bank GTX program was developed at the Fred Hutchinson Cancer Research Center. Donors received G-CSF and dexamethasone. This program treated 19 patients with documented fungal or antibiotic resistant bacterial infections who were either waiting for or recently given HSCT [52]. Adverse reactions occurred in 7 % of the transfusion episodes, with no clear relationship with the presence or development of leukocyte antibodies. Overall, infection resolved in 8 patients. However, none of the 5 patients with aspergillosis cleared their infection.

In another study G-CSF-mobilized GTX collected from related donors were administered to 15 neutropenic patients with hematologic malignancies and fungal infections [53]. Eleven patients had favorable responses and eight of them remained free of infection 3 weeks after therapy.

In a similar study, thirty patients with hematological malignancies received granulocyte transfusions for neutropenia and severe infections during a 12-year period [54]. The donors were given G-CSF and intravenous dexamethasone. A median of 3 transfusions was administered to the patients. For 11 patients (37 %), defervescence and resolution of signs of infection could be attributed to granulocyte transfusions. Mortality at 30 and 180 days after granulocyte transfusions was 40 and 72 %, respectively. No infection-related mortality was reported in patients who responded clinically to the granulocyte transfusions.

A prospective, non-randomized study evaluated the efficacy of granulocyte transfusions for controlling and preventing recurrence of severe infections in patients with hematological malignancies [55]. Fifty-two patients were enrolled between 1997 and 2003, with a total of 67 infectious episodes. The underlying infections were predominantly of fungal origin. In the interventional group, a favorable response was documented in 82 % of the infectious episodes, especially in patients with bacterial infections. In the prophylactic group, no single reactivation of a previous infection occurred. Survival at day 100 after granulocyte transfusions was 64 and 65 % in the interventional and prophylactic group, respectively. With a median follow-up of 3.5 years, 42/52 patients had died, mostly due to the underlying progressive disease.

The RING study is a recently completed randomized controlled study carried out by the NHLBI Transfusion Medicine/Hemostasis Clinical Trials Network which evaluated the efficacy of high-dose granulocyte transfusion therapy [56]. The desired sample size was 236 subjects, in order to have 80 % power to detect a 20 % difference in success rates between the treatment and control groups. Fourteen clinical sites participated and 114 subjects were enrolled. Patients were neutropenic and had a proven/probable/presumed bacterial or fungal infection. Subjects were randomly assigned to receive standard antimicrobial therapy with or without GTX collected from normal donors stimulated with G-CSF and dexamethasone. The median number of granulocytes administered per transfusion was 54.9 × 10^9^. The composite primary endpoint was survival plus a microbial response evaluated 42 days after randomization. The median number of transfusions in subjects randomized to the GTX arm was five. Success rates were 42 % (20/48) and 43 % (21/49) for the granulocyte and control groups, respectively, on intention-to-treat analysis, and 49 % (17/35) and 41 % (16/39), respectively, on per-protocol analysis. Because of low accrual, the power of this study to detect a 20 % difference in the overall success rates was reduced to approximately 40 %. Thus, it cannot be ruled out that a true effect was missed, particularly if the effect is limited to specific patient subsets [56].

A retrospective analysis of 74 patients with refractory hematological malignancies, receiving granulocyte transfusions, showed that patients with documented severe infections might have better survival rates compared with those who do not have severe infection [12]. Patients who died by 12 weeks after granulocyte transfusion initiation were more likely to have leukemia and not to have had recovery of neutrophil counts. Furthermore, the use of G-CSF and IFN-γ as adjuvant therapy were more common in patients who survived the infectious episode. This observation may suggest that the benefits of granulocyte transfusions are greater in the presence of documented severe bacterial or fungal infection. Importantly, these survival benefits were only observed when GTX were administered prior to disease progression and multisystem failure, requiring the use of mechanical ventilation in critical care units.

A recent single-center case series of 11 patients with invasive *Fusarium* infections who were treated with GTX showed a 91 % response rate [57]. Three patients who failed to achieve hematopoietic recovery did not survive, implying that GTX may improve response rates by bridging periods of neutropenia or bone marrow suppression.

Granulocyte transfusions have been combined with G-CSF administration in 56 patients with severe aplastic anemia and severe infections [58]. The median number of granulocyte components transfused was 18; survival at 30, 90 and 180 days were 89, 70 and 66 %, respectively. Among the 31 patients with invasive fungal infections, survival at 30 days, 90 days and 180 days was 87, 58 and 52 %, respectively. Among the 25 patients with refractory severe bacterial infections, survival at 30, 90 and 180 days were 92, 84 and 84 %, respectively. Importantly, survival rate was correlated with hematopoietic recovery. This study suggests that granulocyte transfusions combined with G-CSF could be an adjunctive therapy for treating severe infections in patients with severe aplastic anemia.

It has been shown that accumulation of the transfused granulocytes at sites of infection can help predict the clinical response. In four patients given ^99m^Tc-HMPAO- labeled granulocytes, planar imaging at 1 h (early) and 4 h (delayed) after granulocyte infusion allowed the identification of responders and non-responders based on granulocyte uptake, as assessed by the lesion-to-normal lung ratio. By contrast, granulocyte scintiscans of two patients who were non-responders did not show any granulocyte uptake into the infiltrative lung lesions [59].

The clinical results of the transfusion of granulocytes collected from community donors vs. family donors may be similar [60]. The use of granulocytes collected from community donors bears the advantage of requiring less time to begin the GTX course. In addition, higher increments of the absolute neutrophil count were recorded in patients receiving GTX from G-CSF and dexamethasone-stimulated community donors, compared with patients receiving GTX from G-CSF-stimulated family donors. Overall, 57 and 56 % of patients receiving granulocytes collected from unrelated community and related donors, respectively, had a progressive or fatal course of infection.

Collectively, the available evidence points to the efficacy of GTX as an adjunct treatment modality for severely neutropenic patients who are likely to experience hematopoietic recovery.

## Evidence from the Cochrane Library

The Cochrane Library provides high-quality information based on publication types that are crucial in evidence-based medicine [61]. The Cochrane Database of Systematic Reviews (CDSR) is the primary output of the Cochrane Collaboration. The Cochrane Library differs from PubMed, in that it is a pre-filtered resource that only contains specific publication types (randomized clinical trials/controlled clinical trials in CENTRAL, systematic reviews in Cochrane Database of Systematic Reviews (CDSR) and Database of Abstracts of Reviews of Effects (DARE). The Cochrane Library offers similar search features as PubMed, e.g. usage of MeSH, limiting the search to specific databases or publication dates, saving of searches, and setting up alerts in a personal account.

The Cochrane Collaboration has published two reviews that aim to appraise the literature for randomized controlled trials on granulocyte transfusions for preventing and treating infections in patients with neutropenia or neutrophil dysfunction [48, 62]. In the prevention setting, ten randomized clinical trials were identified that assessed the safety and effectiveness of prophylactic transfusions [62]. Eight trials were undertaken in the US, one in Spain and one in the UK. All the studies but one were published between 1978 and 1987. Donors were given either steroids or no form of medication. G-CSF was used in only one trial, published in 2006. Although the summary results for mortality, mortality due to infection and data on episodes of infection failed to reach statistical significance, there were consistent trends in favor of the intervention [62]. When the trials collecting <1 × 10^10^ granulocytes were excluded, the relative risk ratio was significantly in favor of the intervention. The authors conclude that the review identified a reduction in mortality due to infection in children and a transfusion with >1 × 10^10^ granulocytes. However, the studies were published many years ago and supportive care measures have significantly improved over time. This Cochrane review, first published in 2009, has been recently updated [63]. Twelve trials met the inclusion criteria. One trial was ongoing at time of publication of the updated analysis (https://clinicaltrials.gov/ct2/show/NCT01204788), leaving a total of 11 trials eligible involving 653 participants. Ten studies included only adults, and two studies included children and adults. Overall, the quality of the evidence was judged to be very low or low across different outcomes according to GRADE methodology. All-cause mortality was reported for nine studies (609 participants) and mortality due to infection was reported for seven studies (398 participants). There was no difference in all-cause mortality measured over 30 days between patients receiving prophylactic granulocyte transfusions and those that did not. Similarly, mortality due to infection over 30 days was not different in patients receiving granulocyte transfusions and in those that did not. In the low-dose granulocyte group (<1.0 × 10^10^ granulocytes/day), the number of patients with infection was similar in the two patient groups. However, the number of patients with infection was lower among recipients of intermediate doses of granulocytes (1.0–4.0 × 10^10^/day). Also, the number of patients with bacteremia and fungemia was lower among recipients of prophylactic granulocyte transfusions. This systematic review concluded that there is low-grade evidence that prophylactic granulocyte transfusions decrease the risk of bacteremia or fungemia. Similarly, there is low-grade evidence that the effect of prophylactic granulocyte transfusions is dose-dependent, with doses of at least 1.0 × 10^10^/day being more effective at decreasing the risk of infection. Collectively, there is insufficient evidence to determine any difference in mortality rates due to infection, all-cause mortality, or serious adverse events.

A third Cochrane review included eight randomized clinical trials, published between 1975 and 1984 [48]. Eight studies were conducted in the US, one in Canada; one in Switzerland and one was a multicenter European study. Overall, 149 patients were available for analysis in the intervention arm. In these trials no granulocytes were collected after the administration of G-CSF and/or steroids. The method of granulocyte procurement also differed, being filtration leukapheresis in three studies, discontinuous flow centrifugation in two studies and continuous flow centrifugation in the remaining three studies. The evidence from the eight randomized clinical trials (RCTs) was considered to be inconclusive to support or refute the use of granulocyte transfusions for the treatment of severe infections in neutropenic patients. Although the statistical heterogeneity and clinical diversity of the eight studies may have affected the clinical outcome, there may be a survival benefit for patients administered >1 × 10^10^ granulocytes.

## Alloimmunization following GTX

The efficacy of granulocyte transfusions may be lower in patients with HLA alloimmunization. A retrospective study of alloimmunization to HLA and neutrophil antigens was performed in 18 patients with chronic granulomatous disease (CGD), who had also received repeated granulocyte transfusions. Sera were tested using lymphocytotoxicity, granulocyte agglutination, granulocyte immunofluorescence, monoclonal antibody immobilization of granulocyte antigen, and immunoprecipitation assays. This study showed that sera from 14 of the 18 transfused patients contained WBC antibodies. Seven serum samples reacted in the lymphocytotoxicity, granulocyte immunofluorescence, and granulocyte agglutination assays; seven reacted in the lymphocytotoxicity and granulocyte immunofluorescence assays, but not the granulocyte agglutination assay, and four did not react. Overall, antibodies to neutrophil antigens other than HLA molecules could be detected in sera from eight patients. When the monoclonal antibody immobilization of granulocyte antigen assay was employed, three sera samples reacted with Fcγ receptor III (CD16), three with the 58- to 64-kDa protein carrying the neutrophil antigen NB1, one with CD11a, and one with CD18. In addition, antibodies from three patients were shown to immunoprecipitate a 60-kDa neutrophil protein. Transfusion reactions, including pulmonary toxicity, were documented in 11 of the 14 patients with WBC antibodies, but in none of the 4 patients without antibodies. The patients with WBC antibodies were given a higher number of granulocyte concentrates. This study shows that recipients of granulocyte transfusions often develop alloimmunization and suggests that screening studies for WBC antibodies are indicated periodically during transfusions, after any adverse reactions, or before subsequent transfusion cycles. If WBC antibodies are present, no further granulocyte transfusions should be given unless the granulocytes are collected from HLA- and/or neutrophil antigen-compatible donors [64].

Dihydrorhodamine-123 (DHR) is a marker for cellular NADPH oxidase activity and has been used to monitor the survival of transfused oxidase-positive granulocytes [65]. This technique is based on the ability of normal granulocytes to oxidize the non-fluorescent dye DHR to the fluorescent rhodamine-123 through the respiratory burst. Because patients with CGD have granulocytes that lack NADPH oxidase, any fluorescing granulocytes are of donor origin. Eight out of ten HLA alloimmunized CGD patients receiving granulocyte transfusions experienced adverse reactions, ranging from chills and/or fever to respiratory compromise. The average recovery of oxidase-positive granulocytes was approximately 1 and 20 % in patients with or without HLA allosensitization, respectively. Greater than 1 % in vivo recovery of DHR-enhancing donor granulocytes was correlated with lack of HLA alloimmunization. In five patients, the granulocyte transfusions were discontinued because of severe transfusion reactions. Overall, nine of ten patients cleared the infection. This study suggests that if HLA antibodies are present and the survival of donor granulocytes, as detected by DHR analysis, is low, granulocyte transfusions should be discontinued, as the potential benefits are outweighed by the risks.

## Adverse effects of granulocyte transfusions

Several studies reported high rates of transfusion reactions after the administration of granulocyte concentrates. Severe pulmonary reactions might be attributed to sequestration of the transfused cells into the pulmonary vascular bed. Most transfusion reactions have been documented in patients who were alloimmunized to leukocyte antigens. Anti-leukocyte antibodies could affect post-transfusion increments of neutrophil counts, alter the kinetics of circulating neutrophils and limit the anti-microbial effects of granulocyte transfusions. Importantly, no significant complications from the transfusions were reported in three RCTs that provided compatible leukocytes [66–68].

## Ethical and safety issues related to G-CSF administration to healthy donors

G-CSF effects in healthy volunteers, although normally transient and self-limiting, are currently believed to be more complex and heterogeneous than previously appreciated. In addition to its established role in activating neutrophil kinetics and functional status, G-CSF administration can affect monocyte/DC and lymphocyte numbers and/or function, as well as the hemostatic system [69–71]. In vivo studies have shown that G-CSF levels in the peripheral blood following G-CSF administration peak at 4 h after injection and return to baseline levels after 2 days [20, 72]. Although short-lived, the increase of blood G-CSF levels exceeds the levels that are found in patients with respiratory or bacterial infection [73].

The available clinical data do not provide unequivocal evidence that G-CSF can transform normal hematopoietic stem cells in the absence of predisposing factors. In patients with acute myeloid leukemia (AML) with altered proportions of G-CSF receptor isoforms, G-CSF may promote the survival of leukemic cells. In severe congenital neutropenia and severe aplastic anemia, G-CSF receptor mutations or alterations in the proportions of specific isoforms in some patients appear to render them susceptible to leukemic transformation in the presence of sustained pharmacologic levels of G-CSF [74, 75]. Nevertheless, the theoretical possibility that G-CSF could increase the risk of developing leukemia in stem cell donors exists.

A review of data from >50,000 healthy donors given G-CSF was recently published and documented no evidence for an increased incidence of hematological malignancies [73]. Some studies specifically addressed this issue in granulocyte donors, who may be exposed to multiple doses of G-CSF, repeatedly over subsequent mobilization cycles. In one of these reports, donors who had received G-CSF three or more times for granulocyte apheresis between 1994 and 2002 were matched with control platelet donors for sex, age, and approximate number of donations [76]. Eighty-three donors contributed to 1,120 granulocyte concentrates. With a median follow-up of 10 years, there were seven predefined health events, including malignancies, coronary artery disease and thrombosis, in granulocyte donors and five in platelet donors, suggesting that G-CSF/dexamethasone stimulation is safe. A second paper addressed whether repeated administrations of G-CSF produce monosomy-7 aneuploidy in healthy donors [77]. Chromosomes 7 and 8 were analyzed by fluorescent in situ hybridization (FISH) in CD34^+^ cells from 35 healthy donors after G-CSF administration for 5 days and by spectral karyotyping analysis (SKY) in four individuals to assess chromosomal integrity. The authors also examined 38 granulocyte donors who received up to 42 doses of G-CSF and dexamethasone. No abnormalities in chromosomes 7 and 8 were found in G-CSF-mobilized CD34^+^ cells and no aneuploidy was detected in G-CSF/dexamethasone-treated donors.

The influence of G-CSF on DNA methyltransferase (DNMT) activity and on changes in DNA methylation of candidate genes has been analyzed in peripheral blood cells of 20 healthy unrelated stem cell donors within an observation period of 1 year [78]. The authors performed methylation-specific PCR to detect the methylation status of promoter CpG islands of retinoic acid receptor β (*RAR*-*B*) gene and Ras association domain family 1A (*RASSF1A*) gene. Although DNMT activity increased significantly on the day of donation and 1 day after, baseline values were reached by day +7. In addition, differences in the gene methylation of *RAR*-*B* and *RASSF1A* were not detected between both groups, suggesting no long-lasting increase of DNMT activity or enhanced DNA methylation after G-CSF treatment.

The immunological alterations induced by G-CSF mobilization were prospectively analyzed in 24 healthy donors [79]. Interestingly, platelet, granulocyte, monocyte, B cell, and DC counts, as well as IL-2, IL-8, and IL10 secretion, perturbed at time of G-CSF mobilization, returned to baseline values at 1 month, with T-cell and NK cell counts recovering at 3 months. *In vitro* immunoglobulin production was increased up to 6 months after mobilization. Although some immunologic parameters may be altered in a more persistent manner than initially believed, most alterations remain transient with restoration of normal values by 1 year. We have shown that G-CSF can perturb mitochondrial function and promote T-cell activation-induced apoptosis through the upregulation of Bax [70]. These abnormalities could be counteracted in vitro through the use of an anti-CD95 monoclonal antibody.

A recent report identified 8 longitudinal studies on the incidence of AML among healthy donors mobilized with G-CSF [75]. In aggregate, 40,717 donors provided 151,016 donor-years of follow-up with 3 cases of AML identified, corresponding to an incidence rate of 2 per 100,000 donor-years, based upon the overall reported IR of 3.5 per 100,000 person-years in the United States (SEER data, 2004–2008). However, the latent period of secondary AML is long and the incidence of AML in the general population is extremely low, implying that at least 10 years of follow-up of more than 2000 peripheral blood stem cell donors would be required to detect even a tenfold increase in AML. Thus, an adequately designed and powered study should be performed. It also needs to be considered that HLA-identical sibling donors for patients with leukemia may be themselves predisposed to develop the disease.

Finally, a recent survey on 83 donors who contributed 1120 granulocyte concentrates suggests that G-CSF/dexamethasone stimulation may be safe [76, 80]. This study identified donors who had received G-CSF three or more times for granulocyte apheresis, between 1994 and 2002, and matched them with control platelet donors. There was no difference in blood cell counts between the granulocyte donors and the control platelet donors. Also, no differences were recorded in the occurrence of predefined health events, including malignancies, coronary artery disease, and thrombosis, between the two groups of donors. At a median 10-year follow-up, there were seven such events in the granulocyte donors and five in the platelet donors.

Based on of an assessment of a continuing lack of evidence for an increased risk of malignancy in donors receiving G-CSF, the WMDA issued a statement in 2012, which endorses that, ‘Studies following large numbers of unrelated donors have shown that the risk of developing cancer within several years after the use of G-CSF is not increased compared with donors not receiving G-CSF’ [73]. It is recommended that donors be asked about a family history of leukemia and be offered a long-term follow-up. The statement from WMDA relates to donors who have received ‘originator product G-CSF’ (Neupogen^®^, Filgrastim, Amgen) and does not necessarily apply to other mobilizing agents.

## Cytokines and combination strategies to prevent/treat infections

IL-8 is a pro-inflammatory chemokine that can mobilize hematopoietic stem cells in mice and monkeys. In non-human primates, IL-8 with a dose range of 30–50 μg/kg of body weight induces a 8.7-fold increase of blood neutrophils with peak counts being achieved 45 min after injection. IL-8-mobilized granulocytes were functionally normal, in spite of decreased chemotaxis and adherence abilities, as well as H_2_O_2_ production index. This study suggests that IL-8-induced neutrophils could be used for transfusion purposes [81].

Cytokines such as GM-CSF, M-CSF and IFN-γ have been used to treat specific infections [82]. GM-CSF induced complete clearance of fungal infections in 6 of 8 evaluable patients receiving amphotericin-B and GM-CSF [83]. Responses were similarly favorable in patients with bacterial infections. M-CSF has been used in addition to standard anti-fungal drugs to treat infectious complications after allogeneic HSCT, with responses observed in 6 of the 24 treated patients [84]. A long-term follow-up study of 46 consecutive HSCT recipients at The Fred Hutchinson Cancer Research Center observed a 27 % overall survival in patients given 100-2,000 µg/m^2^ M-CSF from day 0 to 28 after determination of progressive fungal disease, compared with 5 % in 58 similar historical controls [85]. The survival advantage was entirely because of a 50 % survival rate in patients with Candida infection and Karnofsky scores greater than 20 %. M-CSF was well tolerated, although patients receiving higher doses experienced a reduction in platelet counts. IFN-γ has been administered twice weekly to 128 patients with CGD [86]. In this study, the IFN-γ-treated group developed significantly fewer infections compared with patients receiving placebo.

IFN-γ1b has been administered concomitantly with granulocyte transfusions to enhance the host defense against fungal pathogens [87]. In this retrospective study, 20 patients mostly with proven or probable invasive fungal infections received high-dose granulocyte transfusions and a median of 9 doses of IFN-γ1b. Most patients also received G-CSF or GM-CSF during combined treatment with granulocytes and IFN-γ1b. Four weeks after the commencement of therapy, 45 % of patients had complete or partial resolution of infection, whereas another 3 patients (15 %) experienced infection stabilization. The 60 % overall response rate observed in this study is encouraging and prompts the evaluation of this combined therapeutic strategies in patients with invasive fungal infections. Enhancement of the microbicidal activity of granulocytes by IFN-γ1b may be attributed to up-regulation of MHC class II, improved ex vivo survival of granulocytes and increased generation of superoxide through the degradation of intracellular tryptophan.

It has been shown that the elevation of intracellular phosphatidylinositol (3,4,5)-triphosphate signaling with PTEN inhibitor SF1670 can enhance the efficacy of granulocyte transfusions [88]. In mice with thioglycollate-induced peritonitis, intravenously injected SF1670 significantly elevated neutrophil recruitment to the inflamed peritoneal cavity. Furthermore, pre-treatment with SF1670 increased the efficacy of granulocyte transfusions in a mouse neutropenia-associated bacterial pneumonia model, with approximately 40 % of surviving mice after transfusion with SF1670-pretreated neutrophils compared with 10 % of mice transfused with untreated neutrophils. Importantly, pre-treatment with SF1670 also increased the efficacy of transfusion with G-CSF-mobilized neutrophils. SF1670 enhanced fMLP-elicited signaling and reactive oxygen species production in G-CSF-primed neutrophils.

## Concluding remarks

Data regarding efficacy and complications of GTX are still limited in children. A properly designed, randomized controlled trial of GTX in children with clinically relevant endpoints would help resolve the current controversy surrounding the use of GTX in this patient population. The heterogeneity of study populations, types of infection, antimicrobial therapy and dosage of transfused granulocytes, coupled with lack of randomization, power and outcome parameters make it difficult to propose accurate recommendations. In addition, their efficacy may be enhanced by cytokine therapy, including IFN-γ, GM-CSF and G-CSF. Overall, granulocyte transfusions remain an important therapeutic modality in patients with difficult-to-treat opportunistic infections, especially as a bridge towards spontaneous recovery of neutrophil counts in patients who achieve remission of their underlying disease [89].
